# Children with rare diseases: do they really have an increased risk of developing epilepsy?

**DOI:** 10.1186/1824-7288-41-S2-A53

**Published:** 2015-09-30

**Authors:** Chiara Pantaleoni, Enrico Alfei, Stefano D'Arrigo

**Affiliations:** 1 UO Neurologia dello Sviluppo, IRCCS Fondazione Istituto Neurologico Carlo Besta, Milano, 20133, Italy

## 

Symptoms involving the central nervous system (CNS) are frequently part of the variable clinical picture of genetic-dysmorphic syndromes.

Intellectual disability represent the most common associated finding, but epilepsy is very frequently reported, for example in chromosomal abnormalities [1-3].

Seizure risk has been pointed out to be significantly higher in those with chromosomal abnormalities with respect to general population [4-6], and represent an important element to recognize and define for both diagnostic and therapeutic purposes.

Seizures' semeiology, severity and frequency can be highly variable in different syndromes, depending on the type of genetic defect, but even amongst subjects with the same genotype there can be a great variability in clinical manifestations, even intrafamiliarly.

Therefore, within the association between genetic syndromes and epilepsy, we should consider two possible situations:

1) Syndromes in which epilepsy could represent a “specific marker”. In certain conditions the recognition of a peculiar electroclinical phenotype could give a significant contribution to diagnostic definition of the syndrome. The best known example is Angelman syndrome [7,8], in which the electroencephalographic (EEG) pattern could suggest the suspected diagnosis even in infants and toddlers, i.e. before the “classical” clinical and behavioral phenotype becomes evident. But epilepsy can represent a significant clinical marker in other conditions as well, such as Rett syndrome [9], Wolf-Hirschhorn syndrome [10], and other recently defined deletion or duplication syndromes.

2) Syndromes in which epilepsy is reported occasionally. In these conditions the diagnostic value of the electroclinical phenotype is limited, but the correct identification and treatment of the symptom still has an important effect on individual and familial health and quality of life.

Finally in genetic syndromes some special considerations should be made regarding the role of interictal EEG abnormalities and discussing clinical indications of antiepileptic treatment.
